# Swimming Behavior of *Pseudomonas aeruginosa* Studied by Holographic 3D Tracking

**DOI:** 10.1371/journal.pone.0087765

**Published:** 2014-01-31

**Authors:** Svenja M. Vater, Sebastian Weiße, Stojan Maleschlijski, Carmen Lotz, Florian Koschitzki, Thomas Schwartz, Ursula Obst, Axel Rosenhahn

**Affiliations:** 1 Applied Physical Chemistry, Ruprecht-Karls-University Heidelberg, Germany; 2 Institute for Functional Interfaces, IFG, Karlsruhe Institute of Technology, Karlsruhe, Germany; 3 Analytical Chemistry-Biointerfaces, Ruhr-University Bochum, Germany; University of Cambridge, United Kingdom

## Abstract

Holographic 3D tracking was applied to record and analyze the swimming behavior of *Pseudomonas aeruginosa*. The obtained trajectories allow to qualitatively and quantitatively analyze the free swimming behavior of the bacterium. This can be classified into five distinct swimming patterns. In addition to the previously reported smooth and oscillatory swimming motions, three additional patterns are distinguished. We show that *Pseudomonas aeruginosa* performs helical movements which were so far only described for larger microorganisms. Occurrence of the swimming patterns was determined and transitions between the patterns were analyzed.

## Introduction

Motility plays a key role in the life cycle of bacteria and is essential for biofilm formation, biofilm dispersal, chemotaxis, and virulence. Molecular motors drive a flagellum that propels the bacteria and allows swimming at very low Reynolds numbers [1]. De Kerchove and Elimenech demonstrated that initial cell adhesion and surface coverage is enhanced by bacterial swimming motility [2]. Not only initial attachment of bacteria but also biofilm development, maturation and dispersal rely on motility [3]. For *Pseudomonas aeruginosa*, an ubiquitous opportunistic human pathogen, flagellar motility is essential to form biofilms on surfaces and tissues, especially dangerous to patients with cystic fibrosis or severe burns [4], [5]. The response to external stimuli such as chemical substances, light and temperature by motility mediated chemotaxis enables bacteria to adjust to changes in environmental conditions and guides the colonization of favorable habitats. Chemotaxis has been extensively studied [6], [7] with particular focus on its impact on the movement of bacteria [8], [9], [10]. Bacteria show different characteristic motion strategies depending on the flagellation [8], [11], [12], [13]: Run and tumble, run and reverse, run and stop and run and arc. Run and stop and run and arc are special cases, less frequently described. Peritrichous flagellated bacteria, e.g. *Escherichia coli* or *Salmonella typhimurium*, predominantly show the run and tumble mechanism. A run pattern is induced by bundling several flagella around the cell body if the flagellar motor rotates in a counterclockwise (CCW) direction, whereas the tumbling is caused by a change in flagellar rotation in clockwise (CW) direction and the unbundling of the flagella [14]. Monotrichous flagellated bacteria such as *P. aeruginosa* are not capable of tumbling because they only possess a single flagellum at the pole of the cell body. Instead, they swim in run and reverse patterns [9]. A reversal of the flagellar rotation direction from CCW to CW causes a change in swimming direction from forward to backward swimming [11]. Similar to the run and tumble mechanism, the propagation direction is controlled by adjusting the frequency of switching between forward and backward motion. This way of motion is often found in marine bacteria, e.g. *Vibrio alginolyticus* and seems to be more effective in the oceanic environment than the run and tumble strategy [15], [16].

Investigations on the swimming behavior of bacteria are crucial to understand chemotaxis, biofilm formation and virulence. Imaging and tracking of different flagellated bacteria in 2D video microscopy experiments have been the subject of several previous studies [17], [18], [19], [20], [21] and revealed different motion patterns, especially near solid surfaces [21], [22], [23], [24], [25]. Conventional microscopic techniques, however, bring the disadvantage of a shallow focal depth which complicates tracking, particularly in the case of free swimming bacteria, as they frequently swim out of the focal plane. Also, the missing third component of the velocity vector hampers quantitative analysis of the motion patterns. Berg presented the first 3D tracking method for bacteria based on video microscopy using a motorized objective which overcame the problem of a shallow depth of field [26]. Several investigations with this device provided important insights into the 3D movement of bacteria and led to the observation of the run and tumble phases in *E.coli* [8], [27], [28].

Here we apply digital holographic microscopy (DHM), a lenseless 3D tracking technique with the advantage of not being prone to aberration effects introduced by imaging optics and not being dependent on scanning procedures to capture 3D information [29], [30]. For these reasons digital holographic microscopy has found numerous biological tracking applications e.g. tracking of dinoflagellates, algal spores, spermatozoa and trypanosomes [31], [32], [33], [34], [35], [36], [37], [38]. The sizes of these microorganisms range from 4 µm to 25 µm. The tracking of smaller organisms with holographic microscopy is challenging because the resolution of most experimental setups is limited and the scattering contrast caused by the tiny bacteria is small [39]. In our study we show for the first time the capability of a holographic microscope in the in-line geometry to record trajectories of free swimming cells of the small bacterium *P. aeruginosa* with a length of 2 µm and a diameter of 0.5 µm. The most frequently occurring motion patterns are described and we show that bacteria are able to switch between different swimming patterns.

## Materials and Methods

### Bacterial strains

The swimming ability of different strains of *P. aeruginosa* from cell type collections and environmental isolates was tested in motility-tests on semisolid agar plates. The strains tested were P34, P154, P253, VR143/97, SG81, SG81SR, ATCC27853, and PAO01. The strains ATCC27853 and PAO01 originated from the German collection of microorganisms and cell culture (DSMZ, Braunschweig, Germany). The strains SG81 and SG81SR were environmental isolates from technical water-systems and were provided from the University of Duisburg. The strain VR143/97 is a patient isolate described in Riccio et al. [40]. The strains P34, P154 and P253 are clinical wastewater isolates, which were previously identified as P. aeruginosa by ribosomal sequencing, characterised in antibiotic resistance pattern and molecular genotyping. For holographic experiments only the strain P154 was used. All strains were cultivated on Cetrimide-agar (Merck, Darmstadt, Germany) plates. For the experiments single colonies of bacteria were inoculated in Luria-Bertani broth (Merck) and incubated over night at 37°C and 140 rpm.

### Swimming assay using semisolid agar plates

The preparation of semisolid “swimming” agar plates was carried out according to Tremblay et al. [41]. M9-medium, consisting of the following salts Na_2_HPO_4_, KH_2_PO_4_, NaCl and NH_4_Cl (all purchased from VWR Germany) diluted in aqua dest., and the supplements MgSO_4_, CaCl_2_ and glucose-monohydrat were pipetted to 0.3% Bacto agar (BM, Becton) dissolved in aqua dest. The solution was then filled into petri dishes and dried under laminar flow. The semisolid agar plates were used on the same day as preparation. For the motility-tests overnight cultures of the different strains were diluted in fresh LB broth and incubated for additional 2 h at 37°C. 10 µl of this bacterial suspension was inoculated in the middle of a semisolid agar plate and incubated at 37°C for 48 h. Experiments were repeated three times and the reported values represent the average. Error bars reflect the standard deviations.

### Holographic microscopy

**Figure 1:**
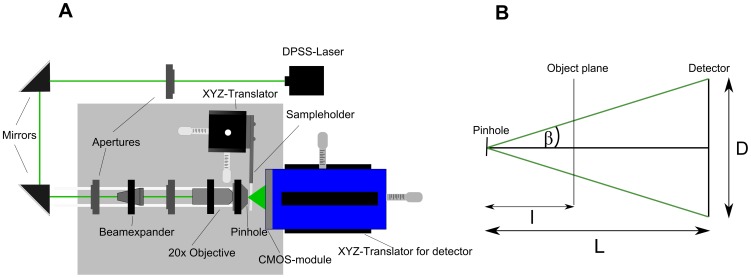
Schematic representations of the digital in-line holographic microscopy setup. (A) The holographic device used in this study consists of the laser source, adjustment mirrors, apertures, a beam expander, the objective, a pinhole and a CMOS camera. B) schematic geometry of the beam path behind the 500 nm pinhole with geometric dimensions relevant for the reconstruction process.

A point source laser holographic microscope in the in-line geometry [29], [30], similar to that utilized for studies with *Trypanosoma brucei* [37], was used in this work to record time series of holograms. The employed setup was composed of the optical components drawn in Figure 1A. A laser beam from a diode-pumped solid-state laser (IMM Messtechnologie, Germany) as a coherent light source with a wavelength of 532 nm and a power of 30 mW illuminated a 500 nm small pinhole (National Apertures Inc., USA) acting as a point source. In order to enhance the photon flux trough the pinhole a 2× Galilean beam expander (Thorlabs, USA) was used to expand the beam before it was focused by a 20× objective (NA = 0.4, Euromex Microscopes, The Netherlands). The small pinhole generated a divergent beam which illuminated objects present in the probed volume. The scattered object wave interfered with the reference wave and formed the hologram on the detector. The detector used was a 10 bit dynamic range pco.1200s CMOS-camera (pco.imaging, Germany; 1280×1024 pixels, 12.3×15.4 mm^2^ chip size, 636 Hz max. frame rate). Each recorded hologram contained 3-dimensional spatial information of the analyzed sample, because amplitude as well as phase information were preserved in the diffraction pattern. By recording a sequence of holograms of moving organisms, a four-dimensional set of coordinates (3 spatial coordinates and time) was generated from which characteristic descriptors for the movement of the organisms were derived. Because of the divergent laser beam the holograph operated as a projection microscope and therefore images of small microorganisms could be magnified. The resolution criterion of the holographic microscope is based on the Abbe limit [42]. It depends on the wavelength of the laser and the numerical aperture (NA), limited by the opening angle of the light cone and the acceptance angle captured by the detector (Figure 1B).

In order to become able to image small bacteria with sufficient resolution, high magnification and strong contrast, the experimental geometry was optimized. The magnification given by M = L/l increased with larger distance between pinhole and the detector. However, this reduced the NA and thus resolution. Higher magnification of the objects in the probed volume can also be achieved by a low distance between pinhole and sample, albeit limited by the thickness of the walls of the cuvette. With the used camera and a distance in the range of 900–1700 µm between pinhole and sample and a distance of 22 mm between pinhole and detector, we found a configuration to achieve a numerical aperture of 0.28 and thus a lateral resolution of 0.95 µm, just sufficient to obtain scattering patterns of bacteria. The resulting field of view had a conical shape due to the divergent nature of the wavefront used for illumination and had the dimensions 0.48×0.48 mm^2^ on the apex plane oriented to the pinhole and 0.83×0.83 mm^2^ on the base plane oriented to the detector.

### Holographic tracking experiments and data analysis

An overnight culture of *P. aeruginosa* strain P154 was diluted with LB broth to an OD_600_ = 0.04 and then incubated at 37°C for 2 h until the bacteria reached the log phase (OD_600_ = 0.2). Immediately before measurement in the holographic microscope, the culture was diluted to reach an OD_600_ = 0.04. As sample cuvettes, biocompatible ibidi μ-slide I Luer channels (ibidi GmbH, Martinsried, Germany) were used with a length of 5 mm, a height of 800 µm and a volume of 200 µl. These channel dimensions allowed observation of *P. aeruginosa* cell suspensions in a large volume tracking of free swimming bacteria with negligible wall effects. The sample cuvette filled with the bacterial suspension was mounted in the light path between the pinhole and the detector. The holographic time series consisting of typically 3000 consecutively recorded holograms were captured with a frame rate of 5 fps. To obtain the real space information and, thus, the object image, the holograms were background corrected and reconstructed using the Kirchhoff-Helmholtz transformation [32], [43], [44]. Because the light passed through objects with different refractive indices such as air, the sample cuvette (Polyethylene) and the medium within the channel, a refractive index correction (RIC) was applied [45]. The reconstructed image stacks were converted into xy-, xz- and yz-projections and trajectories were determined as described in previous work [44]. In general, bacterial motion contains a substantial contribution of diffusion due to Brownian motion [46]. For enhanced perceptibility of the general swimming motions in this article, the extracted x-, y-, and the z-coordinate were smoothed by a local polynomial regression fitting to minimize the contribution of diffusion. The span length of the z-coordinate with 21 data points was slightly higher than the span length of the x- and y-coordinate with 11 data points to account for the worse depth resolution in z-direction compared to the lateral resolution [44]. Most trajectories in the detailed discussion contain both the raw data (as points) and smoothed data by a polymomial regression (solid lines). In the general discussion on pattern classification and transitions between patterns, only the smoothed data are shown. After classifying the patterns, the probability to observe the classified swimming patterns meander, oscillation, helix, pseudohelix and twisting within a given trajectory was analyzed. The reported values are the average probability for each pattern across all 35 trajectories with the corresponding standard error.

## Results

### Motility analysis of *P. aeruginosa* strains on semisolid agar plates

**Figure 2:**
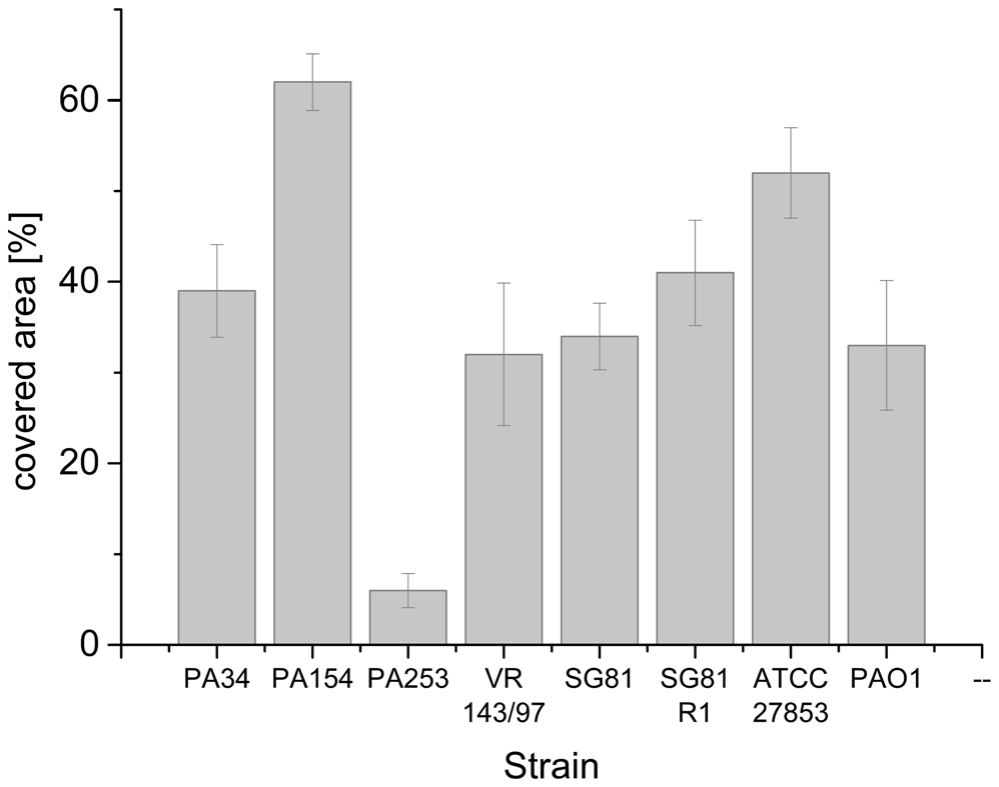
Percentage of covered area on swimming agar for eight different strains of *P. aeruginosa*. The strains were inoculated in the middle of semisolid swimming agar plates and incubated for 48°C in order to investigate the ability for flagellar-mediated swimming. Values represent the mean of three agar plates, error bars are the standard deviations.

To identify a motile strain suited for the tracking experiment, several strains of *P.aeruginosa* were tested on semisolid swimming agar [41]. Therefore 10 µl of a bacterial suspension culture of the strains P34, P164, P253, VR, SG81, SG81SR, ATCC27853 and PAO01 were inoculated in the middle of semisolid swimming agar plates. The plates were incubated for 48 h at 37°C and thereafter imaged with a digital camera (DiMAGE A200, Konica Minolta). The percentage of the covered area in relation to the total area of the plate was calculated from the photographs using the ImageJ software package. As the results in Figure 2 show, the strains ATCC27853 and P154 covered more than 50% of the agar plate and thus could be classified as strong swimmers. The other strains, except P253, with a coverage between 30 and 40% showed pronounced swimming behavior. Only the strain P253 with a coverage of less than 10% demonstrated low swimming activity. Due to the outstanding motiliy of the strain P154, we used this strain for the tracking experiments with the holographic microscope.

### Classification of observed motion patterns of *P. aeruginosa*

**Figure 3:**
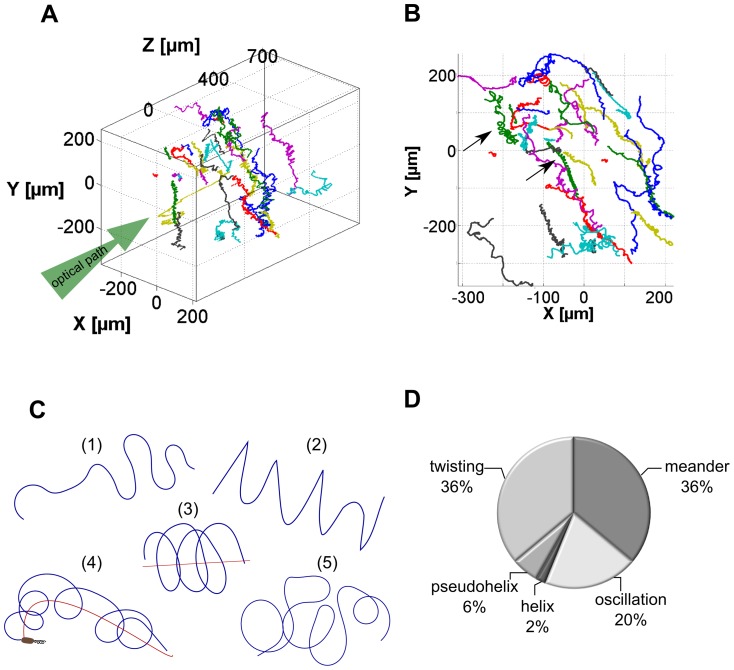
Representation of *P.aeruginosa* trajectories showing different swimming patterns. (A) 3D representation of trajectories of *P. aeruginosa*. The optical path and thus the real space orientation of the 3D cubus is illustrated. (B) xy-projection of trajectories of *P. aeruginosa* as viewed along the optical path. In some trajectories loops can be observed which are marked with black arrows. (C) Schematical representation of 5 different swimming patterns observed for *P. aeruginosa* after tracking of 35 individual bacteria. The different patterns are termed (1) meander, (2) oscillation, (3) helix, (4) pseudohelix and (5) twisting. (D) Probability to observe the classified swimming patterns meander, oscillation, helix, pseudohelix and twisting within a trajectory. Values represent the average over 35 trajectories with the corresponding standard error.

The swimming motility of monotrichous bacteria is known to be very complex and includes a number of orientational changes that occur at different frequencies and involve a range of different swimming motions [18], [22]. Bacteria were cultured in suspension at 37°C for 2 h and bacteria in the log-phase were used in the tracking experiments since it is known that flagellation and motility are optimally developed [47]. The initial three minutes of the holographic movies were reconstructed and 35 trajectories of free swimming *P. aeruginosa* were extracted. The length of trajectories was between 50 s and 3 min, depending on how long the bacterium remained in the field of view. An overview of all 3-dimensional trajectories is shown in Figures 3A–B. The optical path of the holographic microscope is marked to indicate the measurement geometry and it can be seen that gravity induced sedimentation occurred along the vertical axis (Figure 3B). Bacteria were nonetheless homogenously distributed across the observed volume of 0.35 mm^3^ and it became immediately obvious that they showed different swimming patterns. A number of loops are eye-catching in the xy-projection in Figure 3B (labeled with black arrows). These loops gave a first evidence for a helical swimming behavior of *P. aeruginosa*. While not yet measured for bacteria, such helices have been described for larger microorganisms such as protists [48], [49], spores of fungi, spores of plants, and spermatozoa [38].

A close examination of the trajectories resulted in a classification into 5 different swimming patterns schematically represented in Figure 3C which we termed meander, oscillation, helix, pseudohelix, and twisting. The first pattern meander (1) exhibited a smooth and continuous motion with stretched bends and meander-like loops, but without sharp direction changes. In contrast, the oscillatory swimming pattern (2) displayed frequent direction changes with sudden sharp reversals appearing as zig-zag when viewed in a 2D projection. Helical (3) and pseudohelical (4) patterns were characterized by a sequence of consecutive loops. These loops laid on a straight axis for the helical pattern, whereas the axis of the pseudohelical pattern was curved. The last pattern twisting (5) could be described as a sequence of randomly entangled loops with different dimensions and shapes. Also in this case, sharp turns were not observed. Twisting seemed to be an intermediate pattern between helix and meander. The analysis of the probability to observe a specific motion pattern was performed for each trajectory individually. The average occurrences are shown in Figure 3D. More than 70% of the time bacteria swim in the meander or twisting motion. The oscillatory pattern was only found in 19%±5% of the time, while the helical and pseudohelical patterns were seldom observed with a probability of only 2%±1% and 6%±2%, respectively.

### Transition between motion patterns

**Figure 4:**
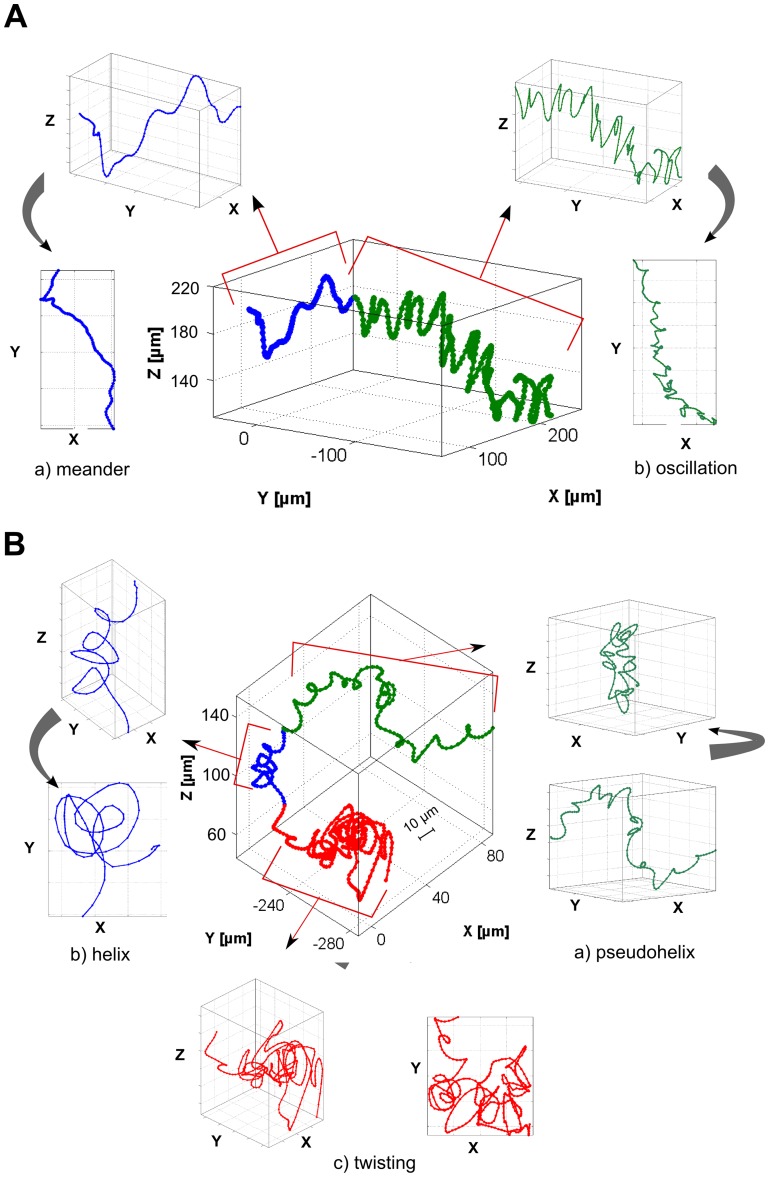
3D trajectories of *P. aeruginosa* with transitions between different motion patterns. (A) Trajectory with a duration of 110 s with switching between the meander and the oscillation pattern. a) and b) illustrate extracted segments of the meander and the oscillation pattern in two different viewing directions indicated by the black arrows. (B) Trajectory with a duration of 130 s with switching between the three different patterns pseudohelix, helix and twisting. The individual segments of each pattern are illustrated in a), b) and c).

The overview in Figure 3 already showed that many trajectories did not consist of a single pattern but changes between the detected motion patterns (Figure 3C) were observed. In 69% of the trajectories at least one transition between different swimming patterns occurred. Figures 4A–B show two examples of trajectories where *P. aeruginosa* clearly switched between motion patterns (the different patterns are marked with a different color). Figure 4A shows a transition from the rather smooth meander pattern (blue) into an oscillatory pattern (green) with frequent, sharp direction changes. The different viewing directions showed that the oscillation occurred in the yz-plane with z being the oscillation direction and y being the propagation direction. Also in other oscillatory segments (not shown here), the main oscillation direction was located in the z-direction, perpendicular to gravity and parallel to the incident light. In Figure 4B the bacterium changed from a pseudohelical behavior (green) into a short helical sequence (blue) and switched then to a twisting behavior (red). While pseudohelical and helical swimming were directed and allowed the bacterium to effectively propagate, the twisting motion showed a strongly reduced directionality. This fact can be calculated for a selected segment as the ratio between straight line velocity (SLV) and curvilinear velocity (CLV), also referred to as linearity of a segment [20]. The SLV was determined as the direct distance from the first point to the last point of the segment divided by its duration. The CLV reflects the length of the trajectory segment divided by the duration of the segment, resulting essentially in the mean velocity. All calculations were done with smoothed data and the mean linearity of four representative segments (not shown except of segments in Figure 4) of each pattern are reported in Table 1. A value of 1 represent a straight-line movement, whereas a value of 0 theoretically denotes movement in a circle. All analyzed patterns had values smaller than 0.5, showing a weak linearity. The twisting pattern has the lowest (0.1±0.02) linearity, and also oscillations reveal small linearity values (0.19±0.04). Pseudohelical (0.27±0.01) and helical motion (0.28±0.04) showed a more pronounced net propagation, while meander seemed to be the most linear pattern (0.42±0.04) observed for *P. aeruginosa*.

**Table 1:** Ratio between straight line velocity (SLV) and curvilinear velocity (CLV) referred to as linearity.

Motion pattern	meander	oscillatory	helical	pseudohelical	twisting
SLV/CLV	0.42±0.04	0.19±0.04	0.28±0.04	0.27±0.01	0.1±0.02

Values are the average and standard deviation obtained from four representative segments.

### The helical swimming pattern

**Figure 5:**
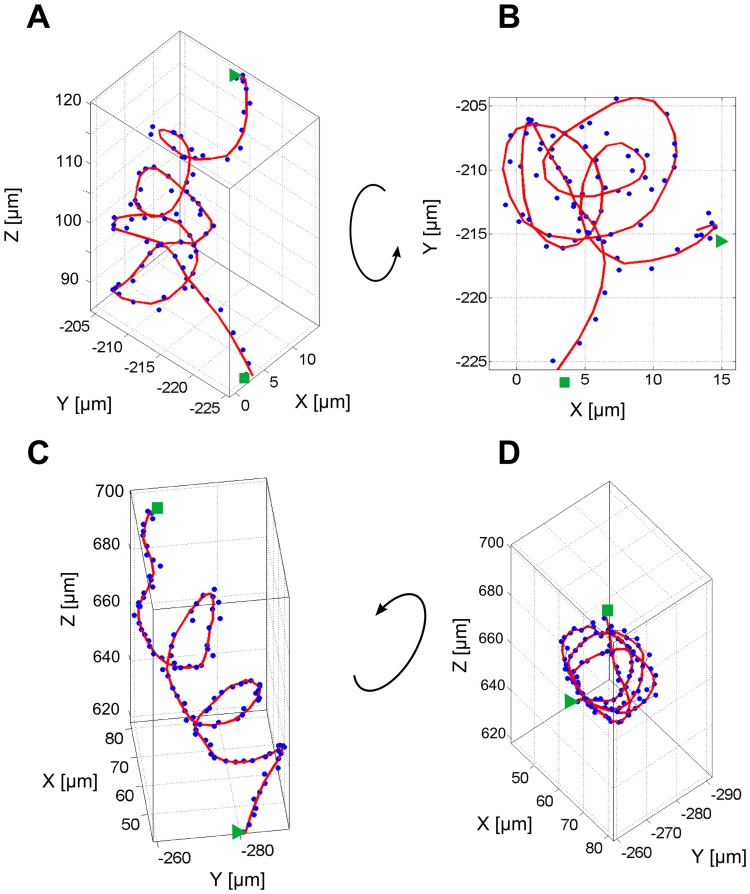
Segments of helical swimming patterns. (A) and (B) 3D representation and view along the helical axis of a right-handed helical segment with 4 loops. (C) and (D) 3D representation and view along the helical axis of a left-handed helix with 3 loops. The start and the end points of the segments are labeled by triangles and rectangles respectively. The blue dots represent the unsmoothed data points, the red line shows the resulting trajectory after smoothing the data with local polynomial regression fitting.

**Figure 6:**
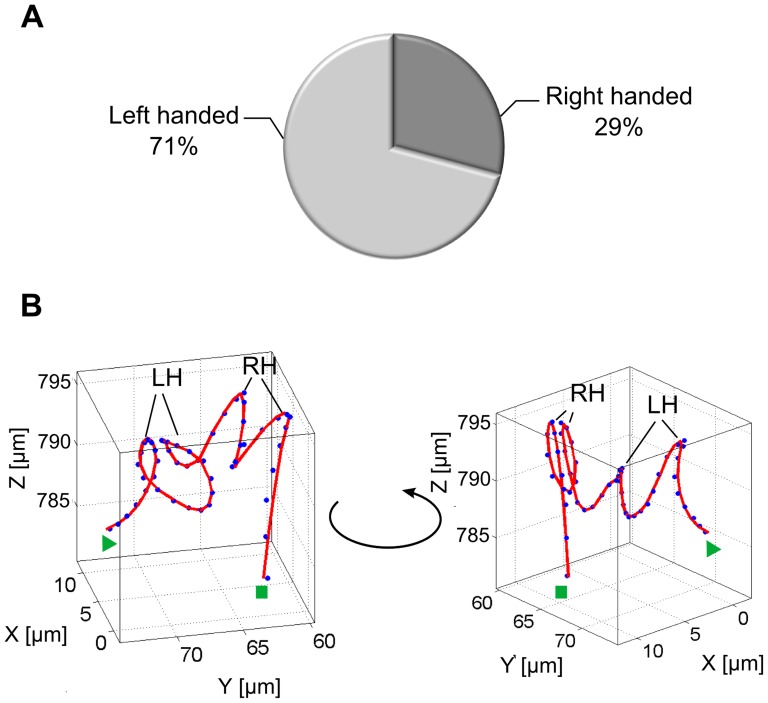
Transition of loop handedness. (A) Percentage of right-handed and left-handed loops determined by analyzing 55 loops in the helical and pseudohelical swimming pattern segments. (B) Extracted segment showing a transition between left-handed (LH) and right-handed (RH) loops. The triangle and the rectangle label the start and the end point of the segment, respectively.

The small probability of only 2%±1% to find the bacterium in a helical motion (Figure 3D) is partly a result of the short duration of this pattern. On average, the helical pattern only consisted of 4 loops strictly along a pronounced axis requiring a swimming duration of ≈20 s (standard deviation 8 s). Figure 5 shows two different examples of helical motions. The start and the end of the segments are labeled by a triangle and a rectangle, respectively. The blue dots represent the raw data in addition to the smoothed red line. A comparison of the two helical segments in Figure 5B and Figure 5D under consideration of their starting point reveals their different handedness. The segment in Figures 5C–D shows a left-handed helix, whereas the segment in Figures 5A–B shows a right-handed helix. While the helical segment in Figures 5C–D exhibits stable loops around the helical axis, the loops in Figures 5A–B have different sizes and shapes and the pitch of the loops is not completely consistent. However, the presence of a sequence of loops around a straight axis gives reason to classify this segment into the helical motion category. To investigate whether the bacteria have a preference to swim either in right-handed or in left-handed loops, the handedness of 55 loops in the helical and the pseudohelical segments was examined. The result in Figure 6A shows a distinct preference (71%) for *P. aeruginosa* to swim in left-handed loops for the helical and pseudohelical motion patterns. Figure 6B shows that different bacteria are able to switch between left-handed and right-handed loops. In the example, the bacterium initially performed two left handed loops and then changed over to right-handed loops. The transition was seamless and no distinct turning point could be observed.

### The oscillatory swimming pattern

**Figure 7:**
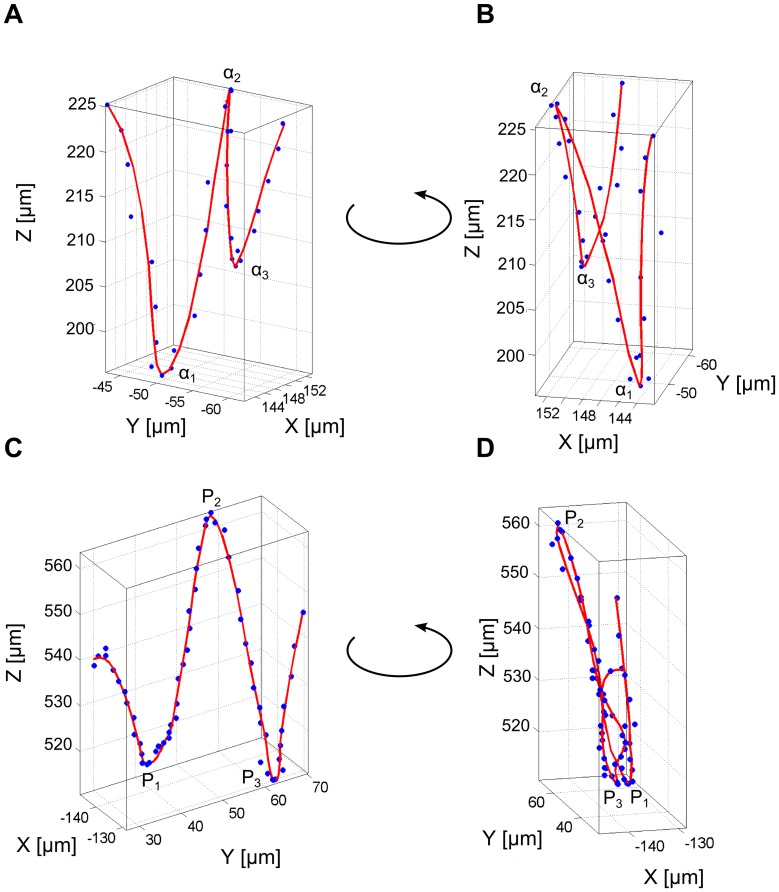
Segments of trajectories showing oscillatory swimming patterns. (A) and (B) Two different viewing direction of a hair-pin-like pattern with short run phases and sharp turning angles α_1_, α_2_ and α_3_. (C) and (D) Two different viewing directions of oscillatory segment with less sharp reversal points P_1_, P_2_ and P_3_.

**Figure 8:**
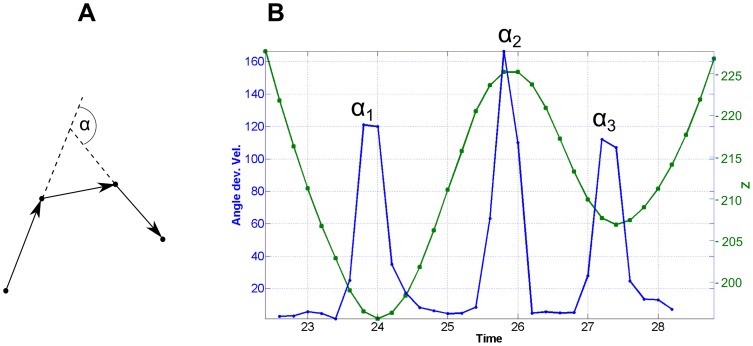
Turning angles of the hair-pin-like pattern. (A) Illustration for the calculation of the turning angles α. The angle is calculated from three consecutive displacement vectors and describes the deviation from a linear motion. (B) Calculated turning angles α (blue) and z-coordinate of the positions (green) of the hair-pin-like pattern in Figures 7A–B.

The oscillatory motion pattern for example in Figure 4A was characterized by highly frequent orientational changes consisting of short run phases followed by sharp turns which were a consequence of reversal of the flagellum and thus reverse the swimming direction. Each flagellar motor reversal induced a transition from forward to backward swimming (forth-back) or from backward to forward swimming (back-forth). The averaged turn frequency calculated from different segments showing the oscillatory pattern was 0.5 Hz (Standard deviation 0.15 Hz). Figures 7A–B demonstrate a short segment of a hair-pin-like pattern which was described earlier for monotrichous bacteria [11], [22]. This pattern involved a very sharp turning angle α which could be almost 180° (α is defined as deviation from linear swimming as sketched in Figure 8A). The very sharp turning angle shown in Figure 8B together with the z-coordinate of the position with a value of α_2_ = 170° was higher than the other turning angles with α_1_ = 110° and α_3_ = 125°. In recent work on *Vibrio alginolyticus* 2-dimensional video microscopy revealed that this bacterium undergoes large directional changes with turning angles of almost 180°, associated with transitions from forward to backward swimming whereas transitions from backward to forward swimming have smaller and more variable turning angles [25]. Figures 7A–B show a very similar behavior for *P. aeruginosa* as the bacterium seemed to perform a transition from backward to forward swimming then from forward to backward, and finally again from backward to forward swimming. This back and forth swimming resulted in the observed oscillation pattern in which sharp and less sharp turning angles alternated. Surprisingly, perfectly sharp turning angles of nearly 180° were rare and in many cases the hairpins were less distinct (Figures 7C–D). As the velocity was lower at the reversal point, usually the density of data points was higher (e.g. points P_1_, P_2_ and P_3_ in Figures 7C–D). Especially when the trajectories were highly sampled, the slow velocity lead to a stronger contribution of Brownian motion and thus a broadening of the hairpin. For the monotrichous bacterium *V. alginolyticus* it could be shown that the forward-backward transitions were faster than the backward-forward transitions [25]. The data point density at the P_2_ reversal point was slightly lower than at the P_1_ and P_3_ reversal points. Considering this, the reversal point P_2_ could be assigned to a forth-back transition and the two other points to back-forth transitions.

## Discussion

Out of a selection of *Pseudomonas aeruginosa* strains, the most motile strain P154 was selected for a quantitative 3D behavior analysis using digital in-line holographic microscopy. Five classes of motion patterns were distinguished: meander, oscillation, helix, pseudohelix, and twisting. While smooth, meandering, and oscillatory behavior were described earlier for 2-dimensional tracking data [11], the twisting, helical and pseudohelical patterns have additionally been identified in this study. We have furthermore found that *P. aeruginosa* is able to switch between the different motion patterns. The transitions were possible between any of the patterns with no preferred order of occurrence. The determination of the switching frequency is hampered by the limited length of the analyzed trajectories (limited observation time) and the used field of view. Latter caused bacteria to enter and leave the field of view at different time points. In some trajectories (31%), bacteria remained in a single motion pattern throughout the experiment and no transition was observed. The longest observed duration of a swimming pattern was 779 data points (≈2,5 min) for the twisting pattern and the shortest duration was 29 data points (≈6 s) for the meander pattern.

Due to their short duration, the helical and pseudohelical patterns were seldom observed but were immediately obvious due to their distinct shape. The loops of the helices could be both, right-handed and left-handed, whereas left-handed loops were observed with a higher probability.

By calculating the linearity of the different patterns, the efficiency to propagate in a net direction and thus the ability to swim towards or away from a stimulus was estimated. For all patterns, this value was below 0.5 and thus motion significantly deviated from an ideal, linear behavior, for which values of 1 are expected. Among all patterns, meandering and helix patterns were found to have the highest values of linearity (0.42±0.04 and 0.28±0.04, respectively) which supports the previous interpretation for other organisms that they serve as highly efficient way of directed movement [49]. A reason for the generally limited linearity for bacterial motion lies in the contribution of Brownian motion that randomly shifts both the position and the orientation of the bacterium [46]. Despite these challenging conditions, bacteria developed ways to readjust their orientation and thus follow a net propagation direction e.g. to perform taxis. In contrast to the run and tumble mode of the peritrichous *E. coli* [8], our results support the previous notion that the backward and forward swimming strategy of the monotrichous *P. aeruginosa* is one way to adjust the orientation for a subsequent directed propagation [11]. Switching between the different swimming modes could be used to adjust the propagation direction e.g. by oscillation and to subsequently switch back to modes that allow enhanced directional stability, such as meandering. This model is supported by the study of Taylor et al. [11] on *P. citronellolis*, showing that the frequency of changes in orientation depended on the presence of attractants or repellents. An increase of attractant led to a directional smooth swimming in which changes in direction were suppressed while a decrease of attractant or increase of repellent resulted in oscillatory movement with highly frequent orientational changes. In our study, the distribution of chemicals in the sample cuvette was rather isotropic. For the future it would be interesting to compare if the presence of attractants and/or nutrients changes the occurrence of patterns. Similar to the previously described oscillatory pattern [11], we distinguished a twisting pattern as characterized by numerous orientation changes and intermediate motion between meander and helix. In contrast to the oscillatory pattern in which orientation changes were connected with sharp reversals shown in Figures 7A–D, the orientation changes in the twisting pattern were caused by loop-like moves. However it can be assumed that the twisting and the oscillatory patterns serve a similar purpose, namely the sampling of the environment and the exploration of favorable locations. If we consider the oscillatory and the twisting patterns as sampling patterns, then the occurrence of such patterns according to Figure 3D sum up to 53% and thus is higher than the occurrence of the directional meander pattern (39%).

Theoretical simulations for the motility of monotrichous, rod-shaped bacteria predict nearly linear helical trajectories for both, forward and backward swimming [50]. For other microorganisms moving at low Reynolds numbers helical motion is known to be the default trajectory [48], [49], [51]. In the case of our study with *P. aeruginosa*, the helical patterns occurred only seldomly (Figure 3D). Why organisms, in our case bacteria, swim in helices is not completely understood. Rotational diffusion and thus the rotation of the cell body around an axis which is not parallel or perpendicular to the translational axis is required to observe helical movement [49]. For *P. aeruginosa* left-handed and right-handed helices were observed and one bacterium is able to switch the handedness of the helix. Left handed helices were slightly preferred (71%). At current we are unable to correlate the handedness of the helices and the swimming mode (forward or backward) of the bacterium as the holographic images do not provide sufficient resolution to detect the flagellum.

In summary, for the first time a full 3D analysis of the swimming motion of *P. aeruginosa* is presented that revealed different motion patterns, transition between the different motions, and their occurrence. The different patterns were analyzed with respect to their directedness and set into a context with the general propagation strategy of bacteria. The work is a first step into understanding the free swimming behavior of *P. aeruginosa* and provides a powerful platform for future attempts to correlate motion with pathogenic and environmental activities. Here, the question arises about the potential presence of different *P. aeruginosa* pheno-groups regarding to their natural origin. Further, the correlation of phenotypical motion strain cluster with virulence properties and/or specific transcriptional activities would be a promising approach to explain the versatility of *P. aeruginosa*. For strain identification or phenotypic characterization we see potential of 3D tracking concerning the quantitative analysis of the distribution of observed motion patterns and the characterization of changes within certain patterns (e.g. diameter of the helices, durations of the swim segments in the run and reverse patterns). As second measure, the occurrence of patterns and the progression of switching patterns would be a powerful phenotype characterization. However, throughout this work it became clear that the duration of some motion patterns can exceed 700 s. Thus, to observe more than one pattern transition in a trajectory and to analyze the transition occurrences, total trajectory durations longer than 5 min would be required. We expect that the additional information provide a useful support of the currently available microbiological diagnostic toolbox. Among the same lines, the work can directly be continued towards research on surface colonization and biofilm formation with relevance for biomedicine and biotransformation processes. Correlation of typical surface exploration patterns with colonization rates and population patterns could aid to identify cues that determine biofilm populations. Especially in such crowded environments, chemotaxis and quorum sensing will become increasingly important, and individual responses on the single organism level to such cues still remains to be understood.
